# RETRACTED: Zhao et al. Analysis of the Molecular Mechanism of Energy Metabolism in the Sex Differentiation of Chickens Based on Transcriptome Sequencing. *Genes* 2024, *15*, 1035

**DOI:** 10.3390/genes16070750

**Published:** 2025-06-27

**Authors:** Ziduo Zhao, Zongyi Zhao, Fufu Cheng, Zhe Wang, Qingqing Geng, Yingjie Wang, Yingjie Niu, Qisheng Zuo, Yani Zhang

**Affiliations:** 1 Jiangsu Province Key Laboratory of Animal Breeding and Molecular Design, College of Animal Science and Technology, Yangzhou University, Yangzhou 225009, China; mz120221557@stu.yzu.edu.cn (Z.Z.); mz120021331@stu.yzu.edu.cn (Z.Z.); mx120230879@stu.yzu.edu.cn (F.C.); mz120231615@stu.yzu.edu.cn (Z.W.); mx120210845@stu.yzu.edu.cn (Q.G.); niuyj@yzu.edu.cn (Y.N.); 006664@yzu.edu.cn (Q.Z.); 2 Joint International Research Laboratory of Agriculture and Agri-Product Safety of Ministry of Education of China, Yangzhou University, Yangzhou 225009, China; 3 College of Biotechnology, Jiangsu University of Science and Technology, Zhenjiang 212018, China; yjwang@just.edu.cn

The journal retracts the article “Analysis of the Molecular Mechanism of Energy Metabolism in the Sex Differentiation of Chickens Based on Transcriptome Sequencing” [1], cited above.

Following publication, concerns were brought to the attention of the publisher regarding a number of figures presented in this article [1].

Adhering to our standard procedure, an investigation was conducted by the Editorial Office and the Editorial Board. While the authors cooperated with the Editorial Office during the investigation, they were unable to satisfactorily explain the irregularities present in Figures 1 and 3. As a result, the Editorial Board decided to retract this publication [1], as per MDPI’s retraction policy (https://www.mdpi.com/ethics#_bookmark30, accessed on 1 June 2025).

This retraction was approved by the Editor-in-Chief of the Genes journal.

The authors agreed to this retraction.
